# Acute Hemolysis and Heme Suppress Anti-CD40 Antibody-Induced Necro-Inflammatory Liver Disease

**DOI:** 10.3389/fimmu.2021.680855

**Published:** 2021-05-12

**Authors:** Marc Pfefferlé, Giada Ingoglia, Christian A. Schaer, Kerstin Hansen, Nadja Schulthess, Rok Humar, Dominik J. Schaer, Florence Vallelian

**Affiliations:** ^1^ Division of Internal Medicine, University of Zurich, Zurich, Switzerland; ^2^ Institute of Anesthesiology, University of Zurich, Zurich, Switzerland

**Keywords:** heme, erythrophagocytosis, anti-CD40, inflammation, hepatitis

## Abstract

Clearance of red blood cells and hemoproteins is a key metabolic function of macrophages during hemolytic disorders and following tissue injury. Through this archetypical phagocytic function, heme is detoxified and iron is recycled to support erythropoiesis. Reciprocal interaction of heme metabolism and inflammatory macrophage functions may modify disease outcomes in a broad range of clinical conditions. We hypothesized that acute hemolysis and heme induce acute anti-inflammatory signals in liver macrophages. Using a macrophage-driven model of sterile liver inflammation, we showed that phenylhydrazine (PHZ)-mediated acute erythrophagocytosis blocked the anti-CD40 antibody-induced pathway of macrophage activation. This process attenuated the inflammatory cytokine release syndrome and necrotizing hepatitis induced by anti-CD40 antibody treatment of mice. We further established that administration of heme-albumin complexes specifically delivered heme to liver macrophages and replicated the anti-inflammatory effect of hemolysis. The anti-inflammatory heme-signal was induced in macrophages by an increased intracellular concentration of the porphyrin independently of iron. Overall, our work suggests that induction of heme-signaling strongly suppresses inflammatory macrophage function, providing protection against sterile liver inflammation.

## Introduction

During hemolysis and in conditions with a localized breakdown of red blood cells (RBCs), erythrophagocytosis and heme-clearance are critical metabolic functions of macrophages (1). This is particularly true for liver macrophages, which are the primary phagocytes that eliminate damaged RBCs during systemic hemolysis (2). Through this archetypical function, macrophages prevent the release of toxic cell-free hemoglobin and heme into the bloodstream or tissues, and iron is recycled to supply erythropoiesis (1–7).

Heme is a disease modifier that may act as a damage-associated molecular pattern (DAMP) and amplify inflammatory responses (8, 9). Once released from cells or hemoproteins, cell-free heme can activate Toll-like receptors (such as TLR4) on the cell surface of leukocytes and endothelial cells, leading to inflammatory signal activation (10, 11). This mechanism can cause endothelial cell activation and vaso-occlusion during a hemolytic crisis with enhanced extracellular heme exposure in sickle cell disease (11–13). The spectrum of hemoglobin and heme triggered adverse physiological effects enforced the evolution of highly effective systems to minimize extracellular hemoglobin and heme exposures (1, 14–17). In most conditions, macrophages internalize damaged but still intact RBCs by erythrophagocytosis before they disintegrate within the circulation (2). Even if intravascular hemolysis occurs, an efficient system of scavenger proteins, including haptoglobin, hemopexin, alpha1-microglobulin, and albumin capture cell-free hemoglobin and heme for subsequent clearance by macrophages and, potentially, other cell types (14, 18–20).

Growing evidence indicates that heme accumulation in macrophages that results from erythrophagocytosis or heme clearance is a suppressor of inflammation and innate immunity (21–25). In chronic genetic hemolytic anemia, erythrophagocytosis profoundly modified the landscape of liver macrophages, skewing their transformation into anti-inflammatory erythrophagocytes that attenuated immune-mediated acute and chronic pathologies in the liver (22). Intracellular heme signaling may also compromise host defense to bacterial infections and adversely affect survival in sepsis (23, 26).

This study investigated how macrophage RBC metabolism and heme may acutely suppress innate immune activation transmitted by non-infectious signaling through CD40 signaling, which induces a macrophage-driven necro-inflammatory liver disease in mice.

## Methods

### Animal Models and Experiments

Wild-type C57BL/6J mice were obtained from Charles River (Wilmington, MA, USA). Tamoxifen-inducible *Hmox1* knockout mice: Hmox1^tm1.1Hes^ were obtained from Harald Esterbauer and crossed with B6.Cg(UBC‐cre/ERT2)1Ejb/J. All mice were housed and bred under specific-pathogen-free conditions at the Laboratory Animal Services Center (LASC) of the University of Zürich. Eight to ten week-old males and females were used in all experiments.

#### Study Approval

All experimental protocols were reviewed and approved by the Veterinary Office of the Canton of Zürich. All animals were treated in accordance with the guidelines of the Swiss Federal Veterinary Office.

#### Phenylhydrazine (PHZ) Induced Systemic Hemolysis

C57BL/6J mice were treated intraperitoneally (i.p.) with 90 mg/kg PHZ hydrochloride. After 12 or 30 h of PHZ injection, blood was removed by heart puncture; livers were collected, digested, and macrophages were purified by magnetic-activated cell sorting (MACS) or anti-F4/80 antibody coated Dynabeads. The concentration of heme was quantified in F4/80^+^ macrophages using the oxalic acid method described by Sassa et al. (27) using oxalic acid purchased from Sigma-Aldrich (#194131).

#### *In Vivo* Injections of Porphyrins

C57BL/6J mice were treated intraperitoneally (i.p.) with 70 µmol/kg of heme-albumin, SnMP, or ZnPP.

#### Agonistic Anti-CD40-Antibody-Induced Systemic Inflammation and Hepatitis *In Vivo* Model

Mice were treated intraperitoneally (i.p.) with 20 mg/kg agonistic anti-CD40 antibody (InVivoPlus, clone FGK4.5/FGK45). After 12 or 30 h of anti-CD40 antibody injection, blood samples were removed by terminal heart puncture for determination of AST/ALT levels (Reflotron AST 10745120, ALT 10745138, Roche) or cytokine measurement (Bio-rad, Bioplex), and the livers excised 48 h after anti-CD40 antibody injection.

### PHZ Preparation

PHZ hydrochloride (Sigma Aldrich, 114715-100g) solutions were prepared by adding 112.5 mg of PHZ to 5 mL PBS. The pH of the solutions was then brought to 7.4, using NaOH 1 M, and 5 mL of PBS was added to a total volume of 10 mL. The solutions were sterile-filtered (0.22 µm) and used immediately.

### Porphyrin Preparation

Hemin (heme-chloride) was obtained from Sigma (#51280), SnMP (tin mesoporphyrin, FSISnM321), MnPP (manganese protoporphyrin, FSISnM321), and ZnPP (zinc protoporphyrin, FSIZn625-9) were all obtained from Frontiers. All porphyrins were dissolved in 10 mL NaOH (100 mM) at 37°C, and then 10 mL of 20% human serum albumin (CSL Behring AG) was added. After 1 h of incubation at 37°C, the pH of the solution was adjusted to pH 7.8, using ortho-phosphoric acid, and the final volume was adjusted to 25 mL with deionized water. The porphyrin-albumin solutions were sterile-filtered (0.22 µm) and used immediately (28).

### Bone Marrow Derived-Macrophage Culture and Stimulation

Bone marrow (BM) cells were obtained from femurs and tibias of 8–10-week-old mice and differentiated in RPMI medium supplemented with 10% FCS and recombinant mouse M-CSF (PeproTech 315-02, lot 0914245) at 100 ng/mL for up to 7 days. At the end of the culture period, the cells were differentiated in BMDMs; 100% of adherent cells were positive for F4/80 antigen as measured by flow cytometry. *Hmox1* tissue-specific deficient BMDMs were treated with 4-hydroxytamoxifen (Sigma H7904) 24 h before starting the experiments.

### Isolation of Liver Macrophages

Mice were anesthetized by intraperitoneal injection of ketamine (80 mg/kg), xylazine (16 mg/kg), and acepromazine (3 mg/kg). Laparotomy was performed to access the portal vein, which was then catheterized for *in situ* liver digestion with 0.4 mg/ml collagenase B buffered solution (Roche, 11088815001). After digestion, the mice were sacrificed, and their livers were excised, mechanically disaggregated in a petri dish on ice, and filtered through a 100 μm pore cell strainer. The cell suspension was subjected to two rounds of centrifugation (60 ×g, 2 min at 4°C), and the pellet was discarded. The supernatants were then centrifuged once more (300 ×g, 5 min at 4°C) to obtain a pellet of nonparenchymal cells containing liver macrophages [protocol modified from (29)].

### Macrophage Enrichment With Magnetic Beads

#### Dynabeads-Based Cell Enrichment

To positively select F4/80^+^ macrophages from liver cell suspensions for mRNA extraction, Dynabeads Sheep anti-Rat IgG (Invitrogen, 11035), in combination with purified Rat anti-mouse F4/80 antibody (BD Pharmigen, 0.5 mg/ml, 565409) and DynaMag™-2 Magnet (Thermo Fisher Scientific, 12321D) were used according to the manufacturer’s protocol for positive selection (Invitrogen). The purity of the separated cells was more than 95% for the F4/80 antigen.

#### Microbeads-Based Cell Enrichement

To positively select F4/80+ macrophages from liver cell suspensions, anti-F4/80 MicroBeads UltraPure (Miltenyi, cat. n. 130-110-443), and magnetic cell separation LS columns (Miltenyi, cat. n. 130-042-401), and the QuadroMACS Separator (Miltenyi, cat. n. 130-090-976) were used for MACS according to the manufacturer’s protocol (Miltenyi). The purity of the separated cells was more than 95% for the F4/80 antigen.

### Flow Cytometry

Macrophages were pre-incubated with LIVE/DEAD Fixable Near-IR cell stain kit (Invitrogen, L34976) and with Mouse BD Fc Block™ (≤ 1 μg/million cells in 100 μL, BD Biosciences #553141) at 4°C for 5 min and then stained with Pacific Blue anti-CD45 (5 µg/mL, BioLegend #109820), anti-CD11b FITC (5 µg/mL, BD Biosciences #557396), anti-CD11b APC-cy7 (2 µg/mL, BioLegend #101226), anti-F4/80 APC (5 µg/mL, BioLegend 123116), or anti-F4/80 PE (2 µg/mL, BD Pharmigen 565410) antibodies. After cells were fixed with formaldehyde 2% and permeabilized with permeabilization buffer (eBioscience, #00-8333-56), ingested erythrocytes were stained intracellularly with anti-TER-119 PE (2 µg/mL, Stemcell #60033) antibody. Stained cells were analyzed using the LSRFortessa (BD) or the SP6800 Spectral Analyzer (Sony). Data were analyzed using FlowJo and FCS express 6 (De Novo software).

### Gene Expression Analysis

#### Microarray Experiments and Data Analysis

Total RNA was isolated using the RNeasy Mini Kit (Qiagen, Hilden, Germany) according to the manufacturer’s protocol, including on-column DNA digestion (RNase-Free DNase Set; Qiagen Hombrechtikon, Switzerland). The quality of each RNA sample was measured on an RNA Nanochip with a Bioanalyzer 2100 (Agilent Technologies), and only high-quality RNA (RNA integrity number > 7.0) was used for gene expression analysis. RNA quantification was performed spectrophotometrically using a NanoDrop ND-1000 spectrophotometer (NanoDrop Technologies, Wilmington, DE, USA). Fluorescently labeled cRNA was produced from 500 ng of total RNA using the Quick Amp Labeling Kit (Agilent Technologies) according to the manufacturer’s protocol. Differential gene expression profiling was performed by competitive dual-color hybridization on whole mouse genome oligo microarrays (mouse: G4846A, Agilent Technologies). Array slides were XDR-scanned and analyzed using the Feature Extraction Software Version 10.7.3.1 (Agilent Technologies). Statistical analysis and visualization were performed using JMP Genomics 7.0 (SAS Institute, Boeblingen, Germany). Full gene array data were submitted to the Gene Expression Omnibus.

#### Real-Time Quantitative PCR

Total RNA was isolated using the RNeasy Mini Kit (Qiagen Hombrechtikon, Switzerland) and reverse transcription was performed with TaqMan reverse transcription reagents (Life Technologies, Basel, Switzerland), according to the manufacturer’s protocol.

Real-time qPCR (RT-qPCR) was performed using the Fast SYBR™ Green Master Mix (Applied Biosystems, 4385612) to determine the expression levels of the target genes listed in **Table 1** below. Relative mRNA levels were calculated using the 7500 Fast System Sequence Detection Software Version 1.4 (Applied Biosystems) and normalized to *Hprt* levels for each experimental sample.

**Table 1:** Primer pairs for the genes analyzed in gene expression analysis.

TARGET GENES	FW primer	REV primer
*Hmox1*	aggctaagaccgccttcct	tgtgttcctctgtcagcatca
*Nqo1*	agcgttcggtattacgatcc	agtacaatcagggctcttctcg
*Slc7a11*	gattcatgtccacaagcacac	gagcatcaccatcgtcagag
*Il6*	gctaccaaactggatataatcagga	ccaggtagctatggtactccagaa
*Tnf*	tcttctcattcctgcttgtgg	gaggccatttgggaacttct
*Il12b*	aaggaacagtgggtgtccag	gttagcttctgaggacacatcttg
*Il1b*	agttgacggaccccaaaag	agctggatgctctcatcagg
*Ifnb*	ctggcttccatcatgaacaa	agagggctgtggtggagaa
*Ifng*	atctggaggaactggcaaaa	ttcaagacttcaaagagtctgagg
*Cxcl9*	cttttcctcttgggcatcat	gcatcgtgcattccttatca
*Cxcl10*	gctgccgtcattttctgc	tctcactggcccgtcatc
*Ccl2*	catccacgtgttggctca	gatcatcttgctggtgaatgagt
*Ccl5*	tgcagaggactctgagacagc	gagtggtgtccgagccata
*Hprt*	cctcctcagaccgcttttt	aacctggttcatcatcgctaa
*Nos2*	gcatcccaagtacgagtggt	ccatgatggtcacattctgc
*Cd40*	aaggaacgagtcagactaatgtca	agaaacaccccgaaaatggt

### Western Blotting

Western blotting was performed using 10 μg samples of total protein per well suspended in Lämmli buffer as previously described (30). The western blot protocol included fluorescence detection of the secondary HRP-labeled antibodies (Amersham, GE Healthcare, Glattbrugg, Switzerland) with the SuperSignal West Femto Maximum Sensitivity Substrate Kit (Thermo Scientific #AG 34095). Images were optimized by adjusting the brightness and contrast of the entire image using Photoshop software (Adobe, Adobe Systems GmbH, Zürich, Switzerland). The primary antibodies were pAb anti-HMOX1 antibody (Enzo Life Sciences #ADI-SPA-895) used at a final concentration of 0.2 µg/L.

### Bio-Plex Cytokine Assay

Concentrations of IL6, TNFα, IL12p40, *CCL2*, CCL4, and CCL5 were determined using the Bio-Plex Cytokine Assays (Bio-Rad). The assay was performed using a Bio-Plex 200 system (Bio-Rad). The results were analyzed using the Bio-Plex Data Pro software (Bio-Rad).

### Metabolic Flux Analysis

Mitochondrial function (oxygen consumption rate) and glycolysis (acidification rate) of BMDMs were measured with a Seahorse XF24 extracellular flux analyzer using the Cell Mito Stress Kit (Agilent Technologies) according to the manufacturer’s instructions.

### Histology

An Olympus IX71 microscope was used for macroscopic imaging of fresh liver and spleen tissue samples. For histological images, the liver and spleen were fixed in 10% formalin for 48 h at room temperature and embedded in paraffin. Microtome sections (5 μm thick) were stained with hematoxylin and eosin (H&E) and photographed using a Zeiss Apotome.2 microscope.

### Statistical Analysis

Data plotting and statistical analyses were performed using GraphPad (Prism 7, San Diego). For comparison of multiple groups, we used analysis of variance (ANOVA) with Tukey’s *post hoc* test. Two-way hierarchical clustering analyses were performed using the Ward algorithm, as provided by JMP14 software (SAS, Germany).

## Results

### Phenylhydrazine Treatment Induces Acute Heinz Body Anemia With Acute Erythrophagocytosis in C57BL/6J Mice

**Figure 1:**
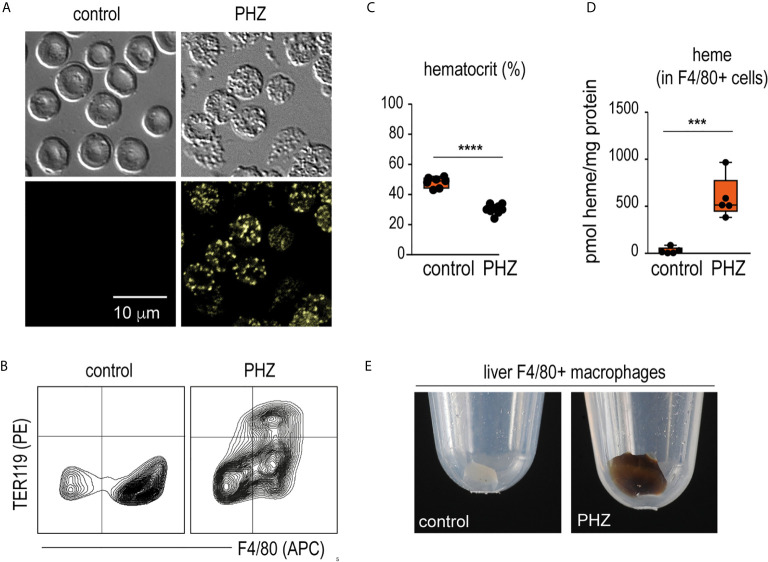
PHZ administration induces acute erythrophagocytosis in F4/80^+^ liver macrophages C57Bl/6J mice were treated with saline (control) or phenylhydrazine (PHZ, 90 mg/kg) and sacrificed 24 or 48 h later for blood and organ collection. **(A)** RBCs of saline- and PHZ-treated C57Bl/6J mice. Upper panels: DIC brightfield image at 600x magnification, lower panels: autofluorescence of Heinz bodies in the green fluorescence channel. **(B)** Representative flow cytometry density plots of liver cells stained for F4/80 and intracellular TER-119 and gated from CD45^+^ liver cells. Data were obtained from a control mouse and a mouse after PHZ treatment. **(C)** Hematocrit of saline- and PHZ-treated mice at 48 h (n = 7–10). **(D)** Heme concentrations in purified F4/80^+^ liver macrophages isolated by positive selection using anti-F4/80 antibody coated magnetic beads, followed by magnetic-activated cell sorting (MACS) from control and PHZ-treated mice (n=5). Heme concentration was normalized to total protein levels in each group. No significant difference in total protein levels was observed between groups. **(E)** Representative photographs of cell pellets of purified F4/80^+^ liver macrophages from saline- and PHZ-treated mice. Individual symbols represent one mouse; ****p < 0.0001 and ***p < 0.001.

Twenty-four hours after a single injection of phenylhydrazine (PHZ), we detected fluorescent Heinz bodies in the circulating RBCs (**Figure 1A**). This was accompanied by extensive erythrophagocytosis of TER119^+^ RBCs by F4/80^+^ macrophages in the liver (**Figure 1B**), followed by a decrease in the hematocrit (**Figure 1C**). Accordingly, F4/80^+^ macrophages isolated from the livers of PHZ-treated animals 48 h after the PHZ injection presented increased intracellular concentrations of heme **(** **Figure 1D**) and the cell pellet had a brown color visible to the eye (**Figure 1E**).

### PHZ Attenuates the Anti-CD40 Antibody-Driven Disease Phenotype by Suppressing Liver Macrophage Inflammation

**Figure 2:**
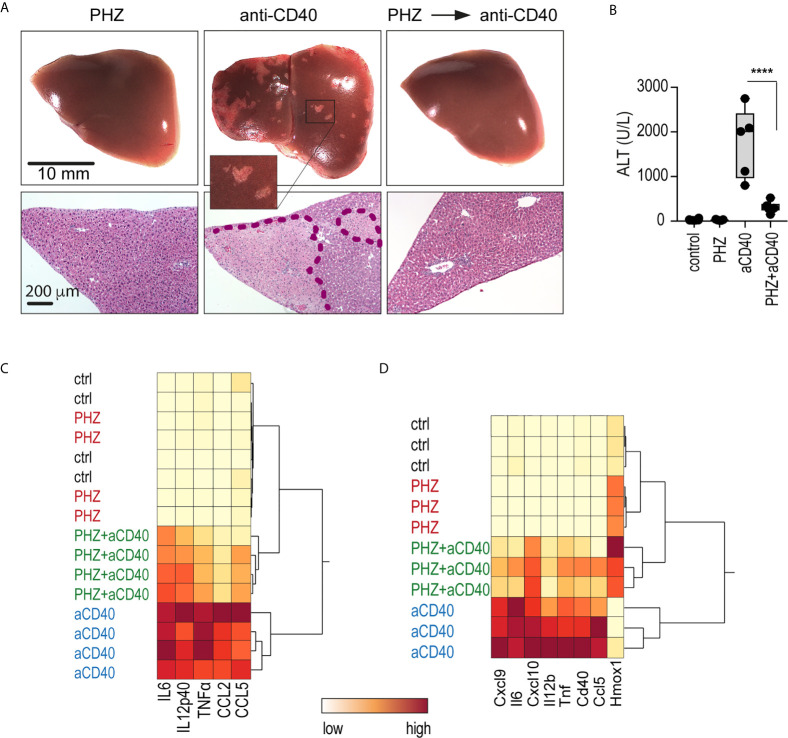
PHZ administration prevents anti-CD40 antibody induced inflammatory liver disease. C57Bl/6J mice were treated with saline (control) or PHZ (90 mg/kg) at T_0h_, 48 h later with anti-CD40 antibody (20 mg/kg) (T_48h_). The animals were sacrificed at T_60h_ for plasma cytokines, at T_78h_ for liver enzymes, and at T_96h_ for liver histology. **(A)** Representative photographs of liver lobes and H&E-stained liver tissue sections. Ischemic infarcts were detected in the anti-CD40 antibody-treated mice (visible as white spots on the liver lobes in the area within the violet dashed line on histology). **(B)** Plasma concentrations of ALT in C57BL/6J mice after treatment with PHZ and/or anti-CD40 antibody (n = 5). **(C)** Heatmap displaying color-coded plasma concentrations of IL6, IL12p40, TNFα, CCL2, and CCL5 in C57BL/6J mice after treatment with PHZ or anti-CD40 antibody (n = 4) (yellow=low expression, red=high expression). **(D)** Heatmap displaying color-coded mRNA expression levels of pro-inflammatory cytokines and Hmox1 from Dynabeads-enriched F4/80 positive liver macrophages in C57BL/6J mice after treatment with PHZ or an anti-CD40 antibody (n = 3) (yellow=low expression, red=high expression). mRNA expression was measured by qRT-PCR. Individual symbols represent one mouse; ****p < 0.0001.

We injected mice with an agonistic anti-CD40 antibody as a model of macrophage-driven inflammatory disease. This treatment induced an acute systemic inflammatory response syndrome with necrotizing hepatitis (31, 32), which was mainly driven by macrophage activation (33). We found that administration of PHZ 48 h before antibody injection suppressed the anti-CD40 antibody-induced disease phenotype, preventing the occurrence of macroscopic and microscopic liver infarcts, and the release of alanine transaminase (ALT) and pro-inflammatory cytokines into the plasma (**Figures 2A–C**). To specifically determine whether PHZ blunts the inflammatory response at the level of liver macrophages, F4/80 positive cells were isolated with Dynabeads following *in situ* liver digestion for 16 h after anti-CD40 antibody administration. **Figure 2D** shows a hierarchical clustering analysis of pro-inflammatory cytokines and *Hmox1* mRNA expression in liver F4/80 positive macrophages of PHZ-treated and untreated animals. A clear segregation was observed in the PHZ-treated and untreated animals in response to anti-CD40, suggesting that PHZ blocked the disease phenotype by repressing macrophage inflammation. No inflammatory response was observed in the macrophages of PHZ-treated animals.

### Porphyrin-Albumin Complexes Deliver Heme Into Liver Macrophages

**Figure 3:**
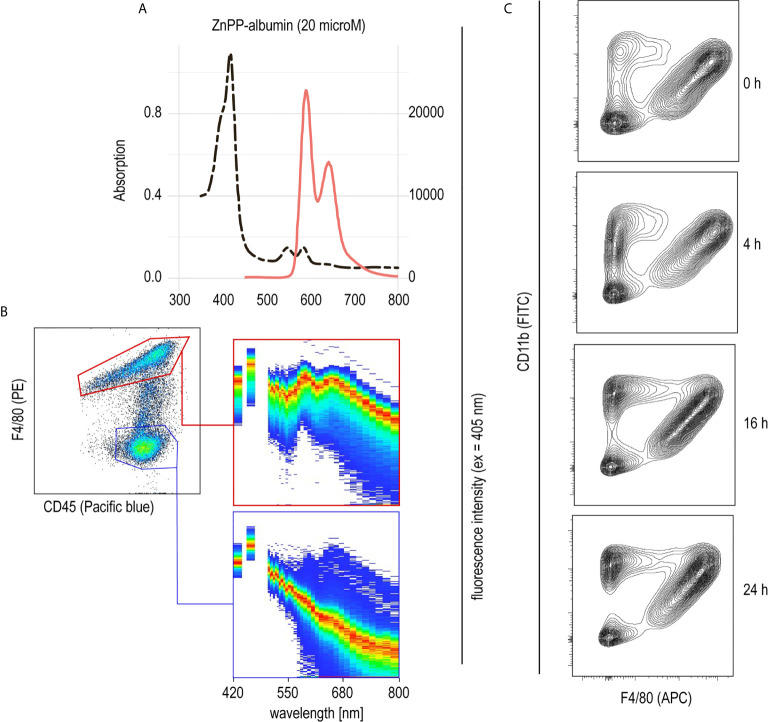
Selective uptake of ZnPP-albumin by F4/80^+^ liver macrophages **(A)** Excitation (dotted black line) and emission (full red line) spectra of ZnPP-albumin excited at 405 nm measured in solution. **(B)** Spectral plots recorded with a spectral cell analyzer for the CD45^+^/F4/80^+^ (red) and CD45^+^/F4/80^-^ (blue) leukocyte populations in a suspension of liver cells from a C57BL/6J mouse treated with ZnPP-albumin. Livers were collected 2 h after the injection. The plots show the cell density on an intensity-wavelength matrix. The red line (area of highest density) indicates the excitation spectrum of the selected cell population. **(C)** Representative flow cytometry density plots of liver single cell suspensions stained for CD11b and F4/80 and gated for live CD45^+^ cells. Livers were collected before (0 h) and 4, 16, or 24 h after heme-albumin injection.

PHZ administration resulted in extensive erythrophagocytosis by liver macrophages and, consequently, high levels of intracellular heme as a result of RBC metabolism. This led us to believe that heme could be the key driver of macrophage suppression, resulting in protection from anti-CD40-induced inflammatory liver disease. To further explore the role of heme in hemolysis-induced macrophage suppression, we established a model to specifically deliver heme into liver macrophages *in vivo*. We have previously described that heme-albumin complexes are useful *in vitro* to induce heme-specific responses in macrophages, while the proinflammatory adverse effects of free heme could be avoided (21, 22). Zinc protoporphyrin (ZnPP) has a fluorescence emission spectrum with two peaks at 591 nm and 647 nm when excited at 405 nm (**Figure 3A**). Using a spectral flow cytometer, we detected the same specific ZnPP emission spectrum in F4/80^+^ macrophages isolated from the liver of mice 2 h after intraperitoneal injection of ZnPP-albumin (**Figure 3B**). In contrast, no ZnPP-signal was recorded in the F4/80 negative leukocyte population, indicating that porphyrin-albumin complexes were preferentially delivered to liver macrophages.

To determine the effects of heme-albumin administration on liver macrophage homeostasis, we characterized liver macrophages by flow cytometry before and 4, 16, and 24 h after heme-albumin injection (**Figure 3C**). Heme-albumin administration induced a time-dependent recruitment of CD45^+^/CD11b^+^ monocytes into the liver. The F4/80^+^ Kupffer cell population remained unaltered, suggesting that the anti-inflammatory effects of heme-albumin described below cannot be explained by macrophage depletion. Collectively, these results suggested that heme-albumin complexes could be used as a vehicle to deliver heme to liver macrophages with minimal adverse toxicity.

### Heme-Albumin Treatment Attenuates the Anti-CD40 Antibody-Driven Disease Phenotype by Suppressing Liver Macrophage Inflammation

To evaluate whether heme could reproduce the anti-inflammatory effects of PHZ, C57BL/6J mice were treated with heme-albumin and, 4 h later, with anti-CD40 antibody. We found that maximum induction of *Hmox1 * and *Nrf2* target genes as surrogate markers of intracellular heme-exposure was reached in liver macrophages four hours after a single injection of 70 µmol/kg heme-albumin (data not shown).

**Figure 4:**
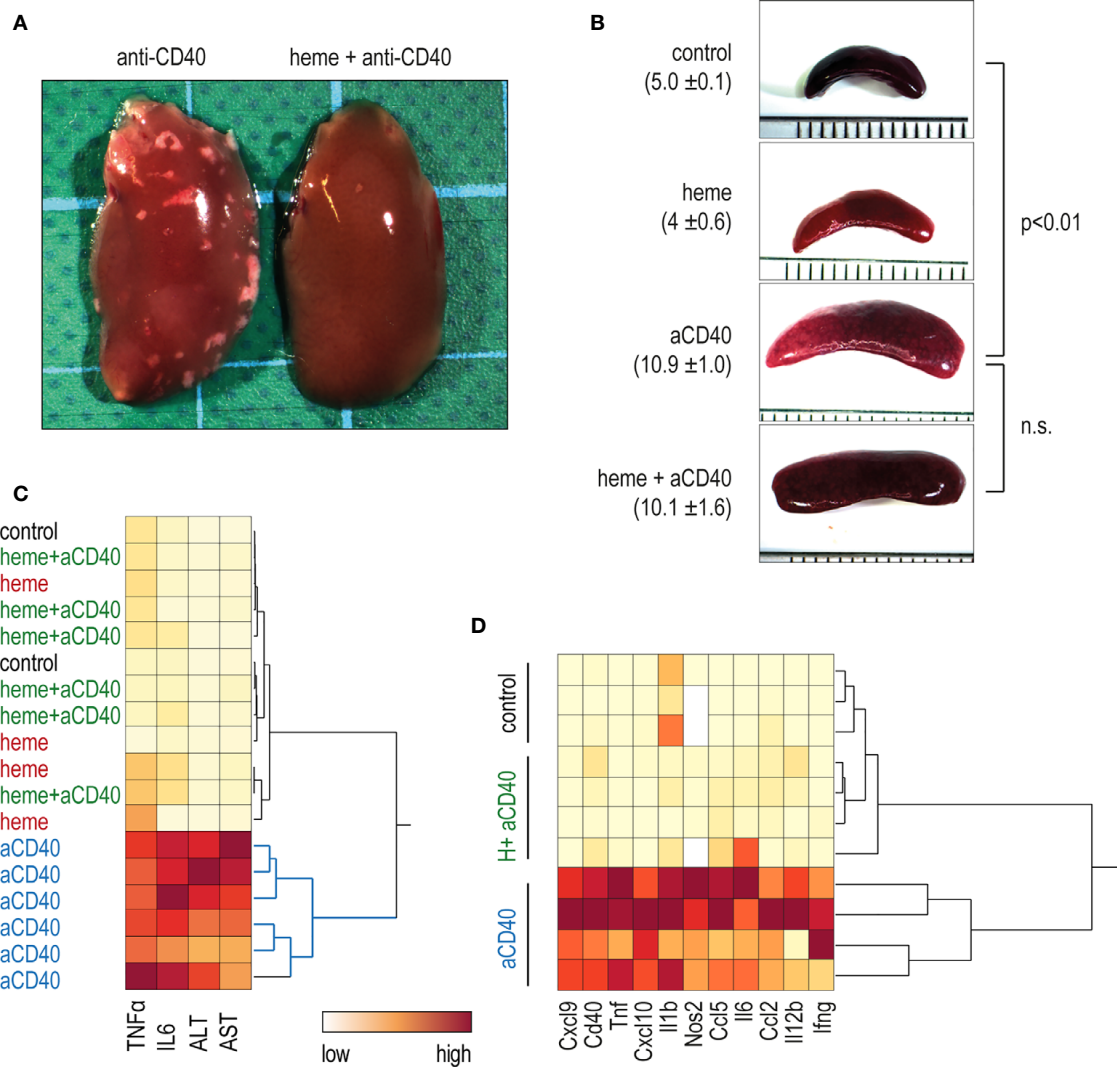
Heme-albumin administration prevents anti-CD40 antibody driven inflammatory liver disease. C57Bl/6J mice were injected with heme-albumin (70 µmol/kg, i.p.) and after 4 h with anti-CD40 antibody (20 mg/kg). The animals were sacrificed at T_16h_ for plasma cytokines or mRNA gene expression, at T_34h_ for liver enzymes, and at T_52h_ for liver histology. **(A)** Representative macroscopic photographs of the liver lobe from mice injected with anti-CD40 antibody, with or without heme-albumin. Necrosis is visible as a white spot. **(B)** Representative macroscopic images of the spleen of mice treated with anti-CD40 antibody, with or without heme-albumin. Displayed is the average size of the spleens (mm) ± 2 SDs. **(C)** Heatmap displaying the color-coded plasma concentrations of IL6, TNFα, and liver enzymes (ALT and AST) in animals treated with heme-albumin (heme) or albumin (control) and challenged with or without anti-CD40 antibody (yellow=low concentration, red=high concentration). **(D)** Heatmap displaying the color-coded mRNA expression levels of proinflammatory cytokines in Dynabeads-enriched F4/80^+^ liver macrophages in C57BL/6J mice that were treated with heme-albumin (heme) or albumin (control) and stimulated with or without anti-CD40 antibody. These data demonstrate that acute heme-albumin treatment mitigated macrophage-driven inflammation and suppressed anti-CD40 antibody mediated disease.

In contrast to saline-treated animals, heme-treated animals displayed no visible liver necrosis (**Figure 4A**). Additionally, based on plasma concentrations of TNFα, IL6, and liver enzymes, the hierarchical clustering analysis in **Figure 4C** shows the clear segregation of heme-and saline-treated animals that were all injected with anti-CD40 antibodies. In contrast, no apparent segregation was visible among the control, heme-treated, and heme+anti-CD40 treated animals. **Figure 4D** shows a clustering analysis of the mRNA expression of proinflammatory genes in liver macrophages isolated with F4/80 antibody coated magnetic Dynabeads 16 h after anti-CD40 injection. Again, a clear dichotomous expression profile was observed in heme-treated and saline-treated animals in response to the anti-CD40 antibody. This indicates that heme specifically suppressed macrophage-driven inflammation, preventing the disease process induced by anti-CD40 in the liver. The enlargement of the spleen, which is a result of CD40-mediated B-cell activation and proliferation (34, 35) was however not affected by heme treatment (**Figure 4B**).

### Macrophage Suppression Is Independent of Heme Iron

**Figure 5:**
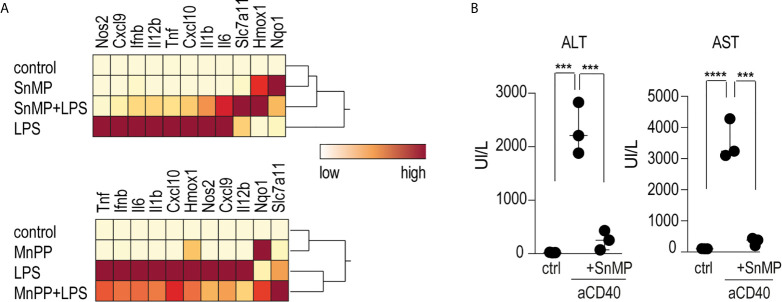
Heme suppresses inflammation independently of iron levels **(A)** Heatmap displaying color-coded mRNA relative expression of proinflammatory and anti-oxidative genes in BMDMs treated with the noniron porphyrin SnMP (top) or MnPP (bottom) for 4 h before stimulation with LPS (10 ng/mL) for an additional 4 h. The data represent the mean expression values from three independent experiments (yellow=low expression, red=high expression). **(B)** Plasma ALT and AST concentrations in C57BL/6J mice treated with or without SnMP-albumin (70 μmol/kg, i.p.) and after 4 h with anti-CD40 antibody for 30 h (20 mg/kg) (n = 3). From these data, we could conclude that metalloporphyrins linked to albumin could replicate the inflammatory suppression of heme-albumin *in vitro* and *in vivo*. ****p < 0.0001, ***p < 0.001 for all panels.

To investigate the role of iron in the anti-inflammatory effects of heme on macrophage-driven anti-CD40 disease, we conducted experiments using the iron-substituted heme-analogs manganese protoporphyrin (MnPP) and tin mesoporphyrin (SnMP). Bone marrow derived-macrophages (BMDMs) incubated with either MnPP or SnMP for 4 h before LPS stimulation showed markedly reduced pro-inflammatory cytokine expression with mild Hmox1 expression (**Figure 5A**). To confirm this finding in mice, we repeated the sequential heme-albumin plus anti-CD40 antibody experiments substituting SnMP for heme-albumin. SnMP is known to have a favorable toxicity profile because it has been extensively studied as a potential treatment for neonatal hyperbilirubinemia (36, 37). SnMP-albumin-treated mice were challenged with anti-CD40 antibody for 4 h. Mice treated with SnMP, but not those treated with saline, were protected from anti-CD40 induced hepatitis, as reflected by the significantly lower plasma transaminase levels (**Figure 5B**).

### Intracellular Heme and Not Its Metabolites Suppress Macrophage Inflammation

**Figure 6:**
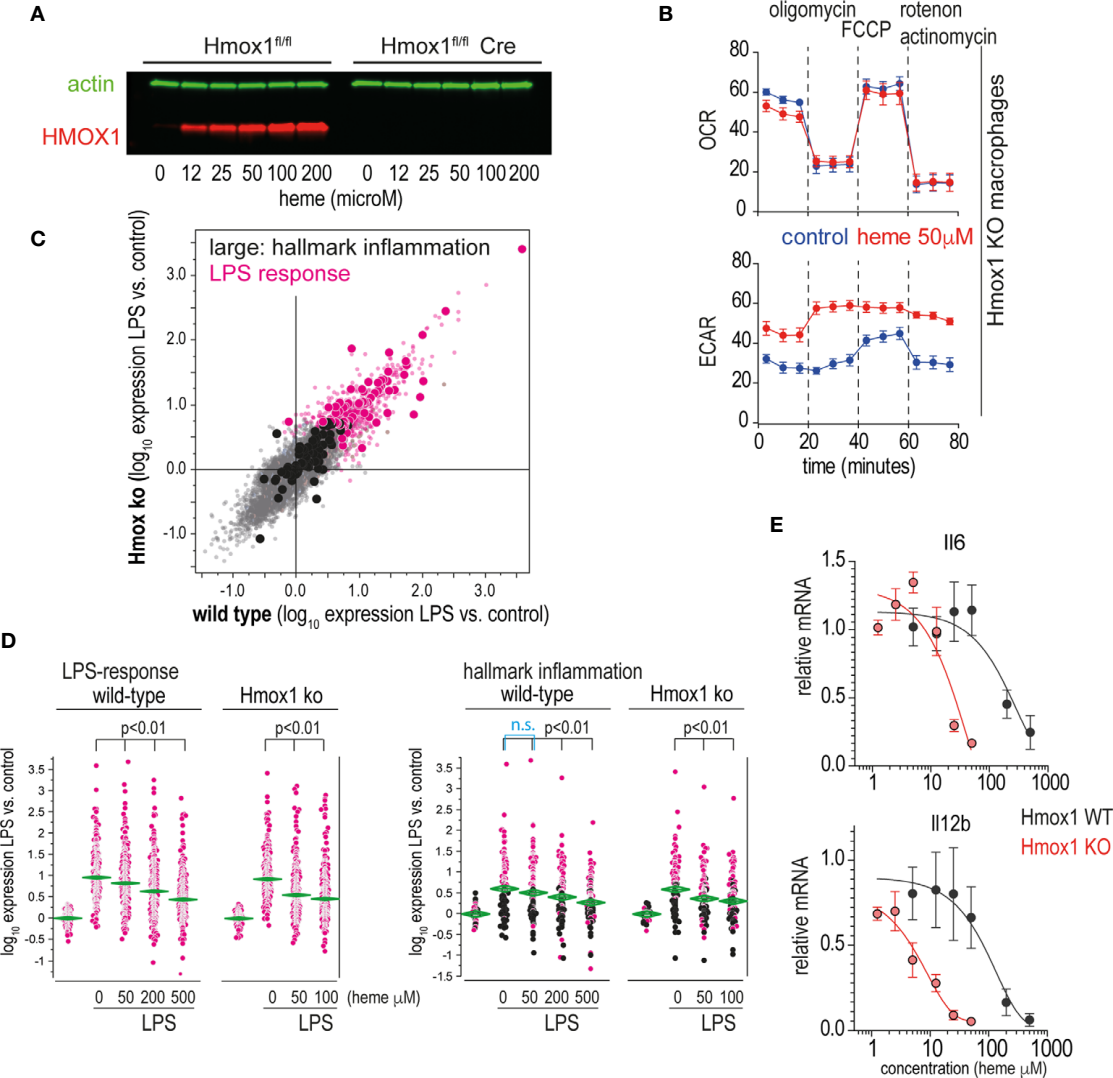
Heme suppresses inflammation independently of its metabolites. **(A)** Western blot of HMOX1 in BMDMs from Hmox1fl/f UBC Cre-ERT2 (knockout) and Hmox1fl/fl (wild-type) mice after exposure to heme-albumin concentrations ranging from 0 to 200 µM for 4 h and LPS treatment for an additional 4 h. Beta-actin was used as a loading control. No HMOX1 expression is visible in the knockout macrophages. **(B)** Oxygen consumption rate (OCR) and extracellular acidification rate (ECAR) of heme-albumin (50 μM)- and albumin (control)-exposed Hmox1 knockout and wild-type BMDMs were measured using a Seahorse XFe24 extracellular flux analyzer. Measurements (n = 3) were recorded according to the standard protocol at baseline, after addition of oligomycin (an inhibitor of ATP synthase), after addition of carbonyl cyanide-4 (trifluoromethoxy) phenylhydrazone (FCCP) (an uncoupling agent, which allows to measure maximum mitochondrial oxygen consumption), and after addition of combined actinomycin A and rotenone (which shuts down mitochondrial respiration). **(C)** Correlation plot of mean mRNA expression changes in wild-type (Hmox1fl/fl) and Hmox1-knockout (Hmox1fl/fl UBC Cre-ERT2) BMDMs that were treated with LPS for 4 h (n = 4 experiments). All genes that were significantly upregulated by LPS are shown in pink (= LPS response). All the genes that define the hallmark “inflammation” gene set in the Molecular Signature Database are depicted with enlarged dots (black and pink). **(D)** Selective presentation of relative expression data for the transcripts defining the “LPS response” and “inflammation” classes across macrophages treated with LPS with/without heme. Statistical analyses of the average expression data were performed by ANOVA with Tukey’s *post hoc* test for multiple comparisons. The data demonstrate a dose-dependent attenuation of the expression of LPS-induced genes with increasing heme-albumin concentrations, and this effect was more pronounced in the absence of Hmox1 expression. **(E)** Relative expression of LPS-induced *Il6* and *Il12b* mRNA in BMDMs from Hmox1fl/fl UBC Cre-ERT2 mice (knockout, red) and Hmox1fl/fl mice (wild-type, black) in response to increasing heme-albumin concentrations. The cells were treated with heme-albumin for 4 h before LPS stimulation. An expression level of 1.0 indicates the mean cytokine mRNA expression in LPS-treated cells in the absence of heme-albumin. The data show the mean ± SD of six biological replicates measured in parallel. n.s., not significant.

Heme levels in macrophages are defined by the flux of extracellular heme into the cell (e.g., *via* erythrophagocytosis or heme-uptake) and by intracellular heme catabolism through heme oxygenases, primarily HMOX1. The observation that the potent heme oxygenase inhibitors SnMP and MnPP (36) had effects on macrophage inflammation that were comparable to those of heme suggested that the anti-inflammatory effects were directly transmitted by the porphyrins and not by metabolites derived from heme breakdown by heme oxygenases. To confirm this hypothesis, we generated an *in vitro* macrophage model with tamoxifen-inducible *Hmox1* deletion. Tamoxifen-treated Hmox1^fl/fl^ UBC Cre-ERT2 macrophages did not produce HMOX1 in response to heme (**Figure 6A**), nor did they show the classical increase in metabolic activity observed in wild-type macrophages metabolizing heme (**Figure 6B**) **(** 38). In the first set of experiments, we compared the response of wild-type and *Hmox1*-deficient mouse BMDMs to LPS-treated mice in the absence of extracellular heme. **Figure 6C** shows the correlation plot of the changes in gene expression induced by LPS treatment for all genes represented on the gene array in the *Hmox1* wild-type (x-axis) and knockout macrophages model (y-axis). These findings demonstrate that the LPS response in the absence of heme is comparable in the two cell types (details of all gene array experiments can be found in the **Supplementary Excel File**).

In subsequent experiments, we analyzed the effects of 50 µM and 200 µM heme-albumin on LPS-induced gene expression. **Figure 6D** shows the effects of heme-albumin on LPS-regulated expression of two defined gene subsets. The ‘LPS-response’ gene set contained all genes that were upregulated in BMDMs by LPS in our experiments. The ‘inflammatory response’ gene set displayed in the right panel is a predefined gene set from the Hallmark Molecular Signature Database (http://software.broadinstitute.org/gsea/index.jsp). The data demonstrate a dose-dependent suppression of inflammatory gene expression by heme, which is more pronounced in Hmox1 knockout cells at lower heme concentrations than in wild-type macrophages. RT-qPCR analysis of LPS-induced *IL6* and *Il12b* mRNA expression confirmed a left-shifted dose-response curve for the heme-suppressive effect in *Hmox1* knockout cells (red line) compared to wild-type cells (black line) (**Figure 6E**). Collectively, these results suggested that heme levels increasing above physiological concentrations during hemolysis determined the anti-inflammatory macrophage response observed in our experiments.

## Discussion

We found that acute PHZ-induced hemolysis leads to an increase in intracellular heme in macrophages, which profoundly suppressed macrophage-driven inflammatory liver disease induced by an agonistic anti-CD40 antibody. This effect of hemolysis was mimicked by the administration of heme-albumin complexes that were specifically endocytosed by macrophages in the liver.

We designed our *in vivo* studies to mimic the two principal pathways macrophages share to internalize hemoglobin and heme: Erythrophagocytosis and endocytosis of hemoprotein-complexes (18, 20, 39). Heinz body anemia is an archetypal example of a disease with extravascular hemolysis driven by excessive erythrophagocytosis of damaged RBCs in the liver (40). We found large numbers of macrophages with internalized RBCs and high intracellular heme levels in the livers of mice following treatment with PHZ. To mimic intracellular heme-exposure in an erythrophagocytosis independent model, we established that heme could be preferentially enriched in mouse liver macrophages after administration of heme-albumin complexes. We confirmed this idea by using ZnPP as a fluorescent heme-analog and spectral flow cytometry, which allowed us to capture ZnPP-specific fluorescence spectra in different cell types of liver single-cell suspensions. Another essential feature of heme-albumin complexes in these experiments was the previous observation in cell-culture that these complexes could deliver heme into BMDMs without triggering pro-inflammatory reactions (21).

To dissect the role of heme on macrophage functions, we selected a CD40-ligation triggered inflammation model with necrotizing hepatitis and cytokine release syndrome. This disease phenotype is strictly driven by macrophage activation (33). Both erythrophagocytosis and heme-albumin complex administration dramatically attenuated the disease pathology, suggesting that the anti-inflammatory block in our experiments occurred at the level of macrophage activation. This was supported by the observation that PHZ and heme-albumin specifically inhibited the anti-CD40-induced pro-inflammatory transcriptional response of liver macrophages.

Experiments with *Hmox1* knockout BMDMs and with *Hmox1* inhibitory heme-analogs (SnMP and MnPP) confirmed that heme, and not one of its metabolites, directly transmits the anti-inflammatory signal in macrophages. The observation that the presence or absence of *Hmox1* dramatically shifted the dose-response curve by which heme suppressed LPS-induced cytokine release in macrophages suggests that HMOX1 might be a key coordinator of inflammatory and RBC disposal functions.

Collectively, our study supports recent reports of anti-inflammatory and immunosuppressive effects of chronic and acute hemolysis. Our group has shown that mice with spherocytosis and chronic hemolysis were resistant to anti-CD40-induced necro-inflammatory liver disease and nonalcoholic steatohepatitis (NASH) (22). In addition, Olonisakin et al. reported that acute hemolysis induced by the transfusion of stressed erythrocytes impaired the innate immune response to Klebsiella pneumoniae infection, aggravating sepsis, and mortality of infected mice (23). Both studies defined an anti-inflammatory and immunosuppressive heme-signaling pathway that includes heme-activation of Nfe2l2/Nrf2 with significant suppression of Stat1 and interferon-regulated genes.

The macrophage-suppressive effects of heme in our studies were profound. It is likely that in addition to Nfe2l2/Nrf2, a synergistic signaling network drives the hypo-inflammatory phenotype in macrophages. Numerous models of infection, sterile inflammation, and transplantation have documented the suppressive function of HMOX1 in innate immunity and inflammation (41, 42). When present in excess, heme, and downstream HMOX1 products might coactively suppress macrophage functions. Other studies demonstrated that heme activates liver-X-receptor (LXR)-coordinated gene expression forcing macrophage differentiation into a phenotype with an anti-inflammatory phenotype and high heme iron-metabolism capacity (43–45). As a central integrator of metabolic and anti-inflammatory signaling, LXR activation might consolidate the effectors of the heme-induced anti-inflammatory macrophage phenotype (46). Heme and other porphyrins can also inhibit the proteasome to directly interfere with the activation of pro-inflammatory NF-kB (47).

RBC-metabolism, iron-recycling, and immune-regulatory functions of macrophages have been characterized in detail, and hypo- or hyperactive failure of either process causes a broad spectrum of diseases such as immunodeficiency, inflammatory liver disease, metabolic diseases, macrophage activation syndromes, anemia, or iron overload (i.e., hemochromatosis) (48–51). However, reciprocal interactions of these archetypical macrophage functions during disease pathogenesis have attracted less attention. Hemolysis with excessive erythrophagocytosis in the liver and inflammatory conditions are both frequent and may co-occur in individuals with a genetic or acquired hemolytic anemia, in patients with sepsis, during blood transfusion, or extracorporeal circulation. Uncovering how hemolysis modulates inflammatory disease processes may help better to understand the heterogeneity of outcomes in those conditions and broaden the spectrum of novel avenues towards personalized disease management.

## Data Availability Statement

The datasets presented in this study can be obtained by contacting Florence Vallelian, Division of Internal Medicine, University Hospital, CH-8091, Zürich, Switzerland. Phone: +41442551697. Email: florence.vallelian@usz.ch.

## Ethics Statement

The animal study was reviewed and approved by Veterinary Office of the Canton of Zürich.

## Author Contributions

MP performed experiments, analyzed data, and wrote the paper. GI performed experiments and analyzed data. CS performed experiments and analyzed data. KH performed experiments. NS performed experiments. RH designed the study and wrote the paper. DJS designed the study and wrote the paper. FV designed the study, performed experiments, analyzed data, and wrote the paper. All authors contributed to the article and approved the submitted version.

## Funding

This study was supported by the Swiss National Science Foundation (MD-PhD scholarship to MP, project 323530_183984, project grant 310030_201202/1 to FV, and 310030_197823 to DJS), and by the Swiss Federal Commission for Technology and Innovation (project 19300.1 PFLS‐LS to DJS).

## Conflict of Interest

The authors declare that the research was conducted in the absence of any commercial or financial relationships that could be construed as a potential conflict of interest.
